# Integration concepts for multi-organ chips: how to maintain flexibility?!

**DOI:** 10.4155/fsoa-2016-0092

**Published:** 2017-03-13

**Authors:** Julia Rogal, Christopher Probst, Peter Loskill

**Affiliations:** 1 Department of Cell and Tissue Engineering, Fraunhofer Institute for Interfacial Engineering and Biotechnology IGB, Nobelstrasse 12, 70569 Stuttgart, Germany

**Keywords:** drug development, flexible multi-organ toolbox, microfluidics, multi-organ chip, organs-on-a-chip, personalized medicine

## Abstract

Multi-organ platforms have an enormous potential to lead to a paradigm shift in a multitude of research domains including drug development, toxicological screening, personalized medicine as well as disease modeling. Integrating multiple organ–tissues into one microfluidic circulation merges the advantages of cell lines (human genetic background) and animal models (complex physiology) and enables the creation of more *in vivo*-like *in vitro* models. In recent years, a variety of design concepts for multi-organ platforms have been introduced, categorizable into static, semistatic and flexible systems. The most promising approach seems to be flexible interconnection of single-organ platforms to application-specific multi-organ systems. This perspective elucidates the concept of ‘mix-and-match’ toolboxes and discusses the numerous advantages compared with static/semistatic platforms as well as remaining challenges.

## Lay abstract

‘Organs-on-a-chip’ are platforms accommodating organ-specific human tissues in microscale 3D chambers with physiologically relevant structure. Broken down to the basic building blocks but simultaneously mimicking essential organ functions, these sophisticated biochips can help reduce the need for animal models in drug development, toxicity screening and basic research. However, to simulate a drug's journey through the human body, it is necessary to consider how a combination of organs responds to a given drug. In this perspective, concepts of realizing such ‘multi-organ platforms’ and the need for ‘mix-and-match’ toolboxes, which contain a range of single-organ units interconnected in individual, application-specific configurations, are discussed.

## Graphical abstract

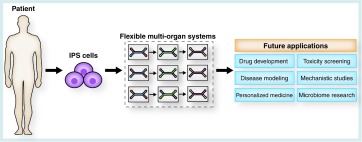

Microphysiological organ-on-a-chip systems integrate human tissue into physiological microfluidic environments recapitulating *in vivo* structure and function. Organs-on-a-chip have become a powerful future alternative to conventional 2D and 3D *in vitro* models and have the potential to significantly reduce and replace animal models employed in the development of pharmaceutical compounds, in toxicological screenings as well as in mechanistic research. In general, organ-on-a-chip systems can be categorized into two concepts (Figure 1A):

**Figure 1.**
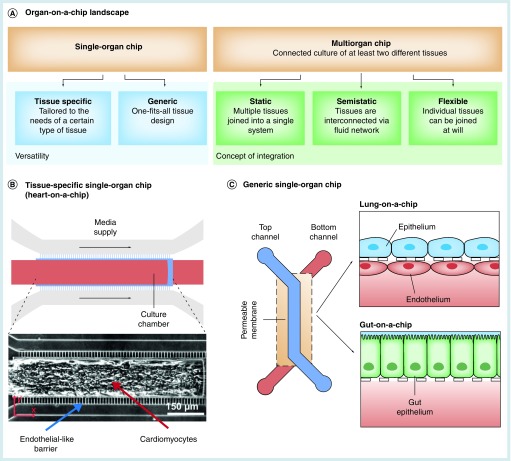
**The general concept of the organ-on-a-chip technology.** **(A)** Current organ-on-a-chip systems can be categorized into two fundamental concepts: single-organ chips integrating one type of tissue or organ only and multi-organ chips featuring at least two different types of tissue or organ compartments. Single-organ systems, in turn, can be subdivided into tissue-specific **(B)** and generic **(C)** single-organ chips. While the geometry of organ/tissue-specific chips is precisely tailored to the needs of a certain type of tissue, a generic one-geometry-fits-all-tissues approach allows a rapid commercialization.

First, single-organ systems integrate one specific type of tissue or organ. Based on their design, single-organ chips can be further subcategorized into organ-/tissue-specific devices and generic platforms. Organ-/tissue-specific platforms (Figure 1B) feature device characteristics that are tailored to one particular type of organ or tissue only. A broad range of single-organ concepts have been developed over the past years including gut [1,2], liver [3–6], lung [7–9], heart [10–14], blood–brain barrier [15,16], brain [17,18], vasculature [19,20] and others [21–24]. Generic single-organ devices (Figure 1C) provide a one-fits-all solution for various types of tissues [3,4]; compared with the platforms with architectures designated to the specific tissue type, these systems rely on a definite geometry convenient for various types of cell cultures and typically work best for barrier tissues.

Second, multi-organ platforms incorporate several tissue compartments into a single device in order to provide a more accurate model of the human body. First attempts to combine multiple tissues have already been made over a decade ago by Shuler and colleagues [25]: their platform features a lung-, liver- and ‘other tissues’-compartment and revealed the enormous potential of multi-organ integration to bridge the gap between animal models and conventional *in vitro* testing systems. Since then, various groups continued developing different multi-organ models [25,26] and investigated, for instance, the methods and effects of adequate organ scaling [27–30].

In this perspective, we will discuss the current concepts for the integration of multiple tissues into a closed multi-organ system. Furthermore, we will consider options revealing how future multi-organ systems could be designed in order to provide more flexibility.

## Multi-organ systems

Multi-organ systems in general aim at the integration of several distinct tissues into a closed fluidic network. Since toxic effects are often not limited to just one organ, but mostly characterized by an intricate cascade of interconnected interorgan events, multi-organ platforms present a promising tool in toxicity screening of pharmaceutical compounds and chemicals. By the envisaged coupling of organ tissues, multi-organ platforms enable a recapitulation of human tissue-tissue interactions related to the drug's – and its associated metabolites – passage throughout the human body. Some compounds, for example, naphthalene [25] or terfenadine [31,32] are not toxic until they are metabolized inside the human body. Moreover, a variety of drugs on the market are sold as prodrugs meaning that they are converted to their actually reactive metabolites only after entering the human body [33,34]. Thus, multi-organ chips have the potential to bridge the gap between preclinical animal testing and clinical trials.

Mimicking the fundamental facets of the human metabolism is best implemented via a media flow through different organ compartments that are physiologically scaled relatively to each other. Thus, the multi-organ chip technology elucidates the consumption, production and exchange of metabolites originating from the drug candidate under testing. Absorption, distribution, metabolism and excretion processes can be dynamically investigated and thereby reveal conclusions on the compound's pharmacodynamics as well as pharmacokinetics. The multi-organ-chip concept is in contrast to the mode of operation of single-organ systems, which are focused on the analysis of direct effects of drug compounds on a specific target tissue. Although the potential of multi-organ-chips is without controversy and there is an urgent need in pharmaceutical, cosmetics and chemical industry, the overall concept of integrating multiple organ chambers and channels into a multi-organ platform to represent tissue/tissue communication and blood circulation has not undergone a groundbreaking change yet; most chips are still limited to implement one specific application at a time.

**Figure 2.**
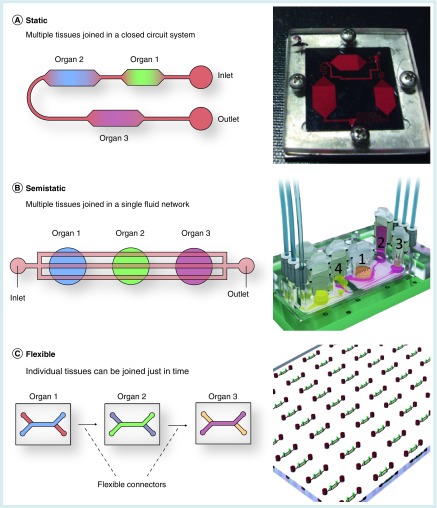
**General approaches for the integration to multi-organ devices.** **(A)** Static systems: multiple tissues are integrated into a single device connected to each other. **(B)** Semistatic systems: tissues are interconnected via a fluidic network with Transwell^®^-based tissue inserts. **(C)** Flexible systems: individual organ/tissue specific platforms are joined together using flexible microconnectors. (A) Reproduced with permission from the Royal Society of Chemistry [38]; (B) Reproduced with permission from the Royal Society of Chemistry [41].

The concepts for the integration of multiple organs into one platform can be categorized into static, semistatic and flexible approaches (Figure 2). Static integration is the most common approach [26,35–39]. Organs or tissues specific for the targeted application are accommodated in chambers located inside a single microfluidic device; the organ compartments are linked to each other via one particular fluid stream. The permanent geometry of the chip confines interorgan connection to a predefined order. The required cells are simultaneously seeded into their respective chambers in the beginning of the experiment and supplied with a common universal medium.

Semistatic concepts, as first described by Wagner and colleagues, are often based on Transwells^®^ with integrated microfluidic channels, pumps and sensors [40]. Individual tissues can be precultured according to their specific needs before integration. Moreover, different combinations of tissues can be tested within a single device [41–44].

Flexible multi-organ systems consist of individual single-organ chips that are connected at any given time using, for instance, micro-connectors [45] or tubings [46]. Thereby, individual organ modules can be loaded and cultured separately using organ-/tissue-specific media. Subsequent to individual culture, the single-organ compartments are then interconnected and henceforth cultured collectively. Flexible multi-organ systems were, for instance, successfully applied to study neuroinflammatory diseases, through the coculture of an endothelial barrier and neural tissue [47].

## A ‘mix-and-match’ toolbox for establishing flexible multi-organ systems

**Figure 3.**
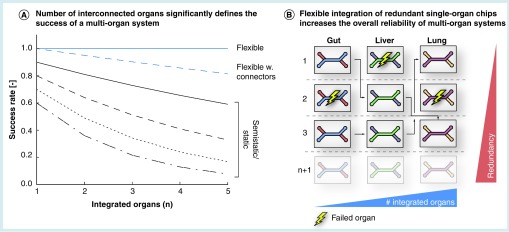
**The concept of flexible multi-organ systems.** **(A)** A comparison of the success rates of multi-organ networks set up according to the three different multi-organ integration concepts. Flexible multi-organ devices theoretically achieve a 100% functionality, irrespective of an increasing number of interconnected organs. In reality, however, we suspect the connectors in between the single-organ compartments to be a minor source of error. Hence, the slight decrease of flexible multi-organ functionality will be caused by the quality and the number of connectors in between the individual organ compartments. In comparison, the success rates of static and semistatic multi-organ systems are significantly lower and diminish with increasing number of interconnected organs. **(B)** The concept of the proposed flexible ‘mix-and-match’ multi-organ tool box intends on preculturing the required single-organ systems separately, and in a parallelized, redundant manner. Upon maturity of all systems, the single units will be connected. By bypassing defective single-organ systems, the performance of the resulting multi-organ system is maintained at its highest level.

Despite the conspicuous progress toward testing pharmaceutical compounds in multi-organ devices, static as well as semistatic multi-organ integration concepts still exhibit a number of restraints on the success rates of those systems. The predefined geometry of the microfluidic chips housing several organ compartments entails two substantial problems: first, the failure of one of the integrated organ chambers consequently and inevitably leads to deficiency of the complete multi-organ system. Second, the fact that culture medium is shared via the connected fluidic network at all time points, from cell/tissue loading until completion of the experiment, limits the tissues’ maintenance due to restrictions in tissue-specific culture medium composition. Additionally, static multi-organ systems require a simultaneous loading of the various incorporated cell types disregarding potential varying differentiation and maturation times of the different types of tissues. As depicted in Figure 3A, the previously discussed aspects result in a significant decrease in the overall chance of functionality of the multi-organ device with an increasing number of integrated organs.

To overcome the limitations brought along with static and semistatic multi-organ integration concepts, we envision the future of multi-organ platforms to lie in the application of a ‘mix-and-match’ toolbox providing single-organ compartments capable of being combined to be tailored to a specific application. For example, when testing a cardiac drug candidate, essential organ systems to be involved in drug testing could be the gut, the liver and the heart. The fundamental idea behind the toolbox system is flexibility. It is achieved by enabling the coordination of organ compartment connection on different levels: in advance to drug testing, individual organ compartments are independently prepared according to tissue-specific requirements, involving distinct culture conditions, media and maturation times. Upon maturity, the individual organ systems will be connected to establish the multi-organ chip and perform the desired experiment.

In the following, we will elucidate in greater detail why, and how, flexible modular multi-organ integration leads to more stable and viable networks compared with semistatic and static approaches.

### Redundancy capacity

Flexible integration approaches offer a redundancy capacity as outlined in Figure 3B. If one of the organ compartments fails, the defect organ compartment can easily be replaced when several single-organ chips of one tissue type are cultured in parallel previous to assembly.

Based on these circumstances, the flexible multi-organ toolbox theoretically assures a full, constant functionality regardless of how many organs are involved into the system (Figure 3A). Given the coordinated maturity of the systems as well as the bypass function for defective organ compartments, flexible multi-organ networks are less predisposed to the previously discussed challenges provoked by increased complexity. However, the conjunction of individual single-organ compartments to the complete multi-organ network raises a few obstacles due to possible failure of connector design and performance; hence, we suppose the overall integrity of flexible multi-organ systems in praxis to be slightly diminished with increasing number of interorgan connections compared with the theoretical optimum.

### Temporal flexibility of the ‘mix-and-match’ multi-organ toolbox

One of the most preponderating advantages of flexible multi-organ systems is the temporal customizability and flexibility regarding the time point of organ compartment connection. In contrast to static multi-organ systems, which require a simultaneous loading of a variety of cell types cultured in the specific organ compartments, flexible multi-organ systems enable a cell seeding that is adaptable to the specific cell type's demands. Since the individual organ compartments will be connected only after the different tissue cultures’ differentiation or maturation, respectively, it is possible to start individual tissue cultures at different time points.

Besides coordination of maturation and differentiation states, the flexible multi-organ concept enables the culture of tissues according to cell-specific protocols, including the utilization of cell- or tissue-specific media until the assembly of the multi-organ system. Among the different types of tissues integrated into multi-organ-chips, the composition of the nourishing media can vary remarkably.

### Freedom in individual organ-chip design & fabrication method

Design and geometry of the tissue chambers play a major role for the organ-on-a-chip technology. Based on the chip's architecture, the mimicked organ's key structures or functional units, respectively, can be emulated whereby immensely affecting the tissue culture's authenticity. The liver-on-a-chip developed by Lee *et al*., featuring biomimetic liver sinusoids that allow hepatocytes to align in canalicular structures [6], for instance, differs significantly in design from the lung-on-a-chip from Huh *et al*., featuring two vertically aligned cell chambers separated by a flexible membrane [7]. Moreover, spatial flexibility allows for more sophisticated 3D coculture systems of multiple cell types. Cellular heterogeneity is an important contributor to the functionality of most organs; using the example of the liver once again, it was shown that a presence of mesenchymal stem cells significantly enhanced the hepatocytes functionality and metabolic activity. This finding is justified by the mesenchymal stem cells’ cytokine secretion that mimics the *in vivo* paracrine signaling and cell–cell interactions leading to enhanced hepatocyte detoxification and synthesis functions [48].

Especially with respect to their 3D designs, the architectures as well as dimensions of organ compartments inside static or semistatic multi-organ system are remarkably interdependent; as these systems are restricted to a single microfluidic platform, fabrication of these systems proves more and more complicated and cost-intensive the larger the variation in the 3D designs of the individual organ compartments. As opposed to this, the only requirement for the individual organ modules of the flexible multi-organ integration approach regarding design issues is an appropriate connection system; the construction of the junctures should be consistent among the organ compartments to be integrated. Apart from that, design aspects of the individual organ modules are completely independent of the other organs’ designs.

### Decoupled individual systems

The geometrical as well as spatial independence among the individual organ compartments comprising a particular toolbox subset enables organ-specific stimulation. The most common stimulation cues applied in organ-on-a-chip systems are of mechanical nature; in this context, a very basic and highly feasible stimulus is the shear force evoked by the flow of fluids through the microfluidic channels. The direct exposure to this shear force is a substantial incentive for the functionality of many types of tissue; for instance, it is an important regulator of endothelial cell functions [49,50]. Another mechanical stimulus frequently integrated into organ-on-a-chip platforms is based on the incorporation of vacuum chambers. This concept is used to mimic physiological breathing in the lung-on-a-chip developed by Huh *et al*.; an artificial alveolar-capillary interface is imitated by exploiting microfluidic concepts and exposing the system to cyclic mechanical strain induced by applying a negative pressure [7]. Another application of integrating vacuum chambers for inducing mechanical strain on cells is used in the gut-on-a-chip system by Kim *et al*., in which human intestinal epithelial cells are exposed to artificially generated peristaltic motions [1]. Indeed, stimulation cues applicable within the scope of the organ-on-a-chip technology are not confined to mechanics; among others, electrical stimulation is frequently employed and proved beneficial for heart-on-a-chip [13] as well as brain-on-a-chip systems [51,52]. Concisely, each individual tissue or organ compartment can retain its unique features despite the increased complexity accompanied by interconnection of the individual organ modules.

### Interlaboratory contribution of existing single-organ systems

Another powerful benefit of the ‘mix-and-match’ multi-organ toolbox is the potential to combine already existing, functioning single-organ systems originating from different laboratories. Upon harmonized interlaboratory coordination, all research groups working on organ-on-a-chip platforms could participate actively in establishing flexible, modular multi-organ platforms.

A cooperation among the groups inevitably entails an interlaboratory adaptation and standardization of the connector system; as long as the inlets and outlets of the chips as well as the connector parts are designed uniformly, interconnecting single-organ units will be conveniently feasible. An appropriate scaling of the individual organ compartments relatively to each other could be solved by merely adjusting the number of replicates of the distinct types of tissue or organ chips.

### Mechanistic modeling with flexible multi-organ systems

Even with regard to mechanistic modeling of drug responses, the ‘mix-and-match’ character of the organ compartment toolbox concept for building flexible multi-organ systems provides various advantages.

First, multi-organ microdevices enable the setup of a customized circulation; in other words, the systems can be adapted to a specific drug, which is supposed to be tested. The fundamental circulation system should feature an organ module recapitulating drug uptake (e.g., gut, lung or skin), an organ module emulating drug metabolism (e.g., liver or adipose tissue) and a target tissue organ module. For example, if a cardiac drug candidate is supposed to be administered orally, the key components of the multi-organ system could include a gut-on-a-chip, a liver-on-a-chip, as well as a heart-on-a-chip. However, it must be taken into account that many other tissues, as for example adipose tissue, may modulate drug function and consequently alter pharmacokinetic as well as pharmacodynamic drug responses. For example, bioaccumulation phenomena in adipocytes were shown to influence the dynamic response of drug testing by drug absorption [25].

Moreover, mechanistic modeling and the setup of a customized circulation allows the integration of diseased-organ models. There are various human disease models-on-a-chip mimicking, for example, cancer [22,53,54], pulmonary edema [8], myocardial failure [55] or neuroinflammation [47]. Especially in combination with the technology of induced pluripotent stem cells (iPSCs), organ-on-a-chip disease modeling is a major advantage in the context of research on rare diseases; one example is the imitation of the cardiomyopathy associated with Barth syndrome, a genetic orphan disease [56,57]. Due to scarcity of the disease, orphan drug development is usually hampered by a lack of subject patients and tissue sources [58].

Additionally, the flexibility of the contemplated multi-organ toolbox system allows a physiologic scaling of the system with correct relative sizes and volumes by adjusting the number of replicates of the involved organ types. As mentioned above, this aspect is of particular importance when interconnecting two or more single-organ platforms that were not scaled relatively to each other; measured against the chip size of the organ representing the smallest volume *in vivo*, the number of replicates of all other single-organ compartments can be determined. While usually a physiologic scaling is desired, nonphysiologic conditions, however, could be beneficial as well; when screening for an unknown toxicity origin, for instance, setting up the volume of a specific organ larger or smaller than *in vivo* could reveal pertinent data on the drug's action, too [59].

Another advantage of the mechanistic modeling with the flexible ‘mix-and-match’ toolbox is the boundless possibilities of interconnections between the organ modules; so far, the focus of multi-organ platforms laid on a serial connection of the tissue or organ units. With the flexible multi-organ approach, the assembly of parallel organ connections or even the generation of feedback loops in the circulation of a drug candidate could be easily implementable, too. Furthermore, the concept would enable a more realistic recapitulation of the human circulatory system; albeit it comprises two separate but closely interconnected systems, namely the cardiovascular and the lymphatic system, the latter of both is frequently overlooked in the organs-on-a-chip technology. These reflections of advanced drug circulation are another important step toward an even more physiological, and therefore more relevant, system.

Besides the connection of the individual organ modules, the flexible toolbox approach further enables a coordinated disconnection of the multi-organ platform after the experiment. This inherent concept of reversibility implements the retrieval of tissue samples after testing; hence, performing conventional analytical methods, as cell culture assays, could be much more convenient than conducting these tests in-chip.

### Direct integration of sensor capabilities

Besides the development of microphysiological environments and the integration of human tissue, the capability to analyze and monitor the tissues and the respective response to drugs or other stimulations is of utmost importance. Analysis of tissues still mainly relies on optical measurement techniques using time-lapse brightfield and fluorescence microscopy in combination with various staining techniques as well as collection of supernatants and tissues samples for analysis with conventional analytical tools. Direct investigation of individual tissues are greatly limited and therefore can only provide a snapshot analysis.

Hence, there is strong demand in the integration of online-sensor capabilities. Already a broad variety of online measurement tools has been integrated to measure crucial parameters such as oxygen, pH [60], glucose and lactate [61]. However, those techniques are mostly based on fluorescent based sensors or thin-film sensors that result in complex fabrications processes along with more cost-intensive and bulky experimental setups, which increase the overall chance of failure. This is especially problematic in static and semistatic multi-organ systems, since the sensors need to be integrated in the platform. The permanent integration also limits the choice of suitable sensors, since especially enzymatic sensors degrade over time. Here, again a flexible toolbox approach provides the possibility to fabricate specific sensor modules and plug them into the system solely when needed.

## Challenges for multi-organ platforms

The enormous potential of flexible multi-organ systems is accompanied by a range of challenges though; aspects to be undeniably addressed in the future include general, technical as well as biological hurdles.

First of all, a standardization of multi-organ systems is required; the overall ambition in this context is to master the balancing act between adequate complexity of the multi-organ system to authentically mimic human (patho-) physiology and simplicity to ensure usability as well as cost efficiency. This involves standardizing the world-to-chip interface properties as well as the interorgan connection system. Moreover, the multi-organ device should be compatible with existing automatization platforms such as pipetting robots, for instance. A standardization step inevitably demands unified protocols for manufacturing, loading and culturing the chips as well as quantitative and qualitative assessment of multi-organ chip functionality. Agreement upon these standardization parameters obligates interdisciplinary and interlaboratory collaboration.

Scaling of integrated system components is a further major challenge. Not only the organ-chip volume but also the media volume and connectors need to be physiologically scaled both compared with their *in vivo* counterpart as well as relative to the other organ-chips. Without an appropriate scaling, residual times of circulating media could be too high or too low falsifying the test results.

Technical challenges coming along with interconnecting individual single-organ systems mainly address issues that threaten the viability and overall stability of the multi-organ network. Air bubbles trapped in the system and contaminations, thereby, rank among the most prevailing contributors to detriments in system stability. Prevention and problem solving toward this entails, for example, the development of appropriate pumps, sealing interconnects, valves and bubble traps, as well as optimized handling properties to maintain sterile conditions.

Likewise, the cost aspect of manufacturing as well as of experiment implementation constitutes a further engineering challenge. In order to hold down costs, basic parts that are in contact with cells, tissue and culture media need to be cheap and disposable while more costly parts, integrating sensors and further electronics, should be designed for reuse.

Among the biochemical aspects faced in flexible multi-organ chip development, composition of a universal culture medium traversing through the different organ compartments presents a major challenge; it is thoroughly demanding to find a medium composition representing the lowest common denominator in supplements content capable of maintaining the heterogeneity of cells [26,27,37,62]. Another biochemical challenge is geared toward the origin of cells embedded into the chips; while many systems still use cell lines or even animal cells, the two main applicable cell sources are primary cells or iPSCs. The most promising potential is frequently attributed to the iPSC technology due to the convenience of obtaining the cells, from skin or blood, for example, the expansion capacity and the reprogramming into any desired cell type. However, the iPSC technology still imperatively requires optimization and standardization of differentiation protocols [63].

## Conclusion & future perspective

Due to the multitude of advantages, multi-organ platforms present a powerful tool in early stages of drug development, personalized medicine and research on human (patho-) physiology. When overcoming the current technical and biological challenges, multi-organ chips assembled according to the flexible ‘mix-and-match’ toolbox concept will be robust, reproducible, reliable and affordable tools in pharmaceutics and medicine.

The process of drug discovery and development, from finding a lead compound to passing the drug candidate on to clinical trials, will be accelerated, less cost-intensive and more predictive; by applying the flexible ‘mix-and-match’ concept, unraveling toxicity effects and drug efficacy will be straightforward and tailored to the specific demands of the drug candidate. Thereby, the dependence on animal models will be significantly decreased and often problematic translational ambiguities between animal and human physiology prevented.

**Figure 4.**
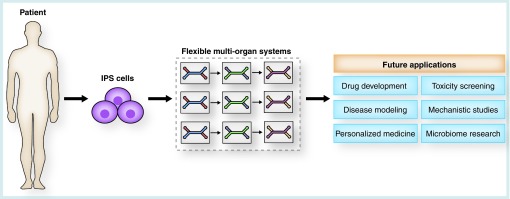
**The future potential of the flexible ‘mix-and-match’ multiorgan toolbox.** In combination with the technology of iPSCs, flexible multi-organ systems will significantly contribute to future advances in a variety of domains of research. The multifarious application areas of the multi-organ system will include drug development and toxicity screening, disease modeling and mechanistic studies, as well as personalized medicine and research on the human microbiome. iPSC: Induced pluripotent stem cell.

Moreover, we envision the elucidated flexible multi-organ concept to especially preponderate in the field of personalized medicine (Figure 4). In combination with iPSC technology, which provides a tool to first reprogram somatic cells from any adult patient to a pluripotent state and then differentiate them into any desired cell type [64–66], ‘patient-on-a-chip’ systems can be constructed. By exploiting autologous cell sources, it will be possible to compile a personalized drug response profile, providing accurate insights into a patient's medication tolerability and therapy outcome. Furthermore, disease-specific cells can be derived from patients. Thus, in-chip patient-specific disease modeling will be feasible and reveal the enormous potential of the organ-on-a-chip technology with regard to discovery of mechanisms and therapies of rare diseases such as degenerative disorders [64]. In addition to personalized drug testing and disease modeling, the abundancy of iPSC sources comes along with the opportunity to study differences among different populations including gender, age or demographics, for instance [58].

In order to fulfill the variety of distinct applications and demands held ready for multi-organ systems, a broad spectrum of different systems will be essential. These systems will range from highly complex low-throughput to miniaturized high-throughput platforms, all of them finding their individual niche in the different stages of drug development, toxicity screening, personalized medicine, disease modeling and further applications. Together, the technology will help to significantly reduce the use of animal models in the coming years and may lead to an animal-testing free R&D in the distant future.

**Executive summary**

**Multi-organ systems**

- Multi-organ platforms incorporate several tissue compartments into single devices in order to provide a more accurate model of the human body.
- Toxic effects are often not limited to just one organ but mostly characterized by an intricate cascade of interconnected interorgan events.
- The concepts for the integration of multiple organs into one platform can be categorized into static, semistatic and flexible approaches.

**A ‘mix-and-match’ toolbox for establishing flexible multi-organ systems**

- Static as well as semistatic multi-organ integration concepts still exhibit a number of restraints on the success rates of those systems.
- We envision the future of multi-organ platforms to lie in the application of a ‘mix-and-match’ toolbox providing single-organ modules capable of being combined to be tailored to a specific application.
- Flexible modular multi-organ integration leads to more stable and versatile networks due to its redundancy capacity, temporal flexibility, freedom in design and fabrication, decoupled individual units, potential to integrate sensors, amenability for mechanistic modeling and openness to interlaboratory exchange.

**Challenges for multi-organ platforms**

- The enormous potential of flexible multi-organ systems is accompanied by a range of challenges.
- Future challenges include but are not limited to conceptual aspects such as standardization or scaling, technical aspects such as bubble prevention, sealing of interconnects or sterility and biochemical aspects such as media composition.

**Future perspective**

- Multi-organ platforms have the potential to be a paradigm shift for a variety of fields of applications such as drug development, toxicological screening, personalized medicine, as well as disease modeling and mechanistic research.
- Flexible integration concepts will be a major factor for the interconnection of individual organ chips to multi-organ systems.
- Depending on the application and requirements in terms of throughput and complexity a wide range of different systems is necessary.
